# The Effects of Montmorency Tart Cherry Concentrate Supplementation on Recovery Following Prolonged, Intermittent Exercise

**DOI:** 10.3390/nu8070441

**Published:** 2016-07-22

**Authors:** Phillip G. Bell, Emma Stevenson, Gareth W. Davison, Glyn Howatson

**Affiliations:** 1 Department of Sport, Exercise and Rehabilitation, Faculty of Health and Life Sciences, Northumbria University, Newcastle upon Tyne NE1 8ST, UK; phillip.g.bell@northumbria.ac.uk; 2 GSK Human Performance Lab., Brentford, Middlesex TW8 9GS, UK; 3 Human Nutrition Research Centre, Institute of Cellular Medicine, Faculty of Medical Sciences, Newcastle University, Newcastle upon Tyne NE2 4HH, UK; emma.stevenson@ncl.ac.uk; 4 Sport and Exercise Sciences Research Institute, Ulster University, Jordanstown, Newtownabbey BT37 0QB, UK; gw.davison@ulster.ac.uk; 5 Water Research Group, School of Environmental Sciences and Development, Northwest University, Potchefstroom 2520, South Africa

**Keywords:** recovery, strenuous exercise, muscle damage, prunus cerasus, functional foods

## Abstract

This study investigated Montmorency tart cherry concentrate (MC) supplementation on markers of recovery following prolonged, intermittent sprint activity. Sixteen semi-professional, male soccer players, who had dietary restrictions imposed for the duration of the study, were divided into two equal groups and consumed either MC or placebo (PLA) supplementation for eight consecutive days (30 mL twice per day). On day 5, participants completed an adapted version of the Loughborough Intermittent Shuttle Test (LIST_ADAPT_). Maximal voluntary isometric contraction (MVIC), 20 m Sprint, counter movement jump (CMJ), agility and muscle soreness (DOMS) were assessed at baseline, and 24, 48 and 72 h post-exercise. Measures of inflammation (IL-1-β, IL-6, IL-8, TNF-α, hsCRP), muscle damage (CK) and oxidative stress (LOOH) were analysed at baseline and 1, 3, 5, 24, 48 and 72 h post-exercise. Performance indices (MVIC, CMJ and agility) recovered faster and muscle soreness (DOMS) ratings were lower in the MC group (*p* < 0.05). Additionally, the acute inflammatory response (IL-6) was attenuated in the MC group. There were no effects for LOOH and CK. These findings suggest MC is efficacious in accelerating recovery following prolonged, repeat sprint activity, such as soccer and rugby, and lends further evidence that polyphenol-rich foods like MC are effective in accelerating recovery following various types of strenuous exercise.

## 1. Introduction

Prolonged, field-based, intermittent sprint sports are popular across the world at both elite and recreational levels [1]. Sports, such as soccer, field hockey and rugby, require a high volume of energy turnover and eccentric muscle actions resulting in metabolic and mechanically induced stress. Indeed, soccer play results in elevated post-match inflammatory [2,3], oxidative stress [2,4,5] and muscle damage [2,4] markers, as well as decrements in physical performance [2,4,5]. The Loughborough Intermittent Shuttle Test (LIST), which closely simulates activity patterns, and the physiological and metabolic demands of soccer [6], has been shown to incur similar stress responses [7,8,9], and, thus, provides a tool to induce similar physiological stress to game-play, but in a controlled environment. In light that athlete schedules require training or competition on multiple occasions within a few days, the importance of recovery strategies when preparing for the next game or training session is critical [10]. This is especially pertinent given that recovery is incomplete at 48 h following actual [2,11] and simulated [9] match-play.

Montmorency tart cherries have been shown on numerous occasions to be of benefit in exercise recovery [12,13,14,15,16], which have been proposed to be as a result of the high concentrations of phytochemicals, and in particular, the flavanoids anthocyanins [14,17,18,19]. These compounds can reduce oxidative stress and been shown to be a cyclooxygenase inhibitor (COX), to a similar extent as NSAIDs [20,21]. A series of studies have investigated the use of Montmorency cherries in influencing recovery from running [14,22], heavy eccentric contractions [15,16] and cycling [12,13]. Collectively, these lines of investigation suggest that Montmorency cherries could also be applied to aiding recovery following high intensity concurrent sports that incorporate and very high metabolic component that is accompanied by high intensity eccentric contractions. 

Given that Montmorency cherries have been shown to be of benefit in exercise recovery following high intensity eccentric contractions and metabolically challenging exercise, it makes the expectation tenable that it could be applied to sports of a concurrent nature. Therefore the aim of this study was to investigate the effect of Montmorency cherries on recovery indices following a protocol designed to replicate the physiological demands of prolonged intermittent sprint activity such as those seen in field based sports. It was hypothesised that MC supplementation would attenuate post-exercise inflammatory and oxidative stress responses, and aid the return of functional performance.

## 2. Methods

### 2.1. Participants

Sixteen semi-professional (step 5 and above in the Football Association National pyramid, UK), male soccer players (mean ± SD age, height, mass, predicted V̇O_2max_ was 25 ± 4 years; 180.8 ± 7.4 cm, 81.9 ± 6.6 kg, 54.9 mL·kg^−1^·min^−1^, respectively) volunteered to take part in the study. All procedures were granted Ethical clearance by the University’s Research Ethics Committee prior to testing and were conducted in accordance with the Helsinki Declaration. Inclusion criteria required participants to have trained in soccer consistently across the preceding 3 years and be free of any lower limb injury for the preceding 6 months. This was assessed through the completion of training history and health screening questionnaires. Following both verbal and written briefings on the requirements of the study, written informed consent was collected from all participants.

### 2.2. Study Design

**Figure 1:**
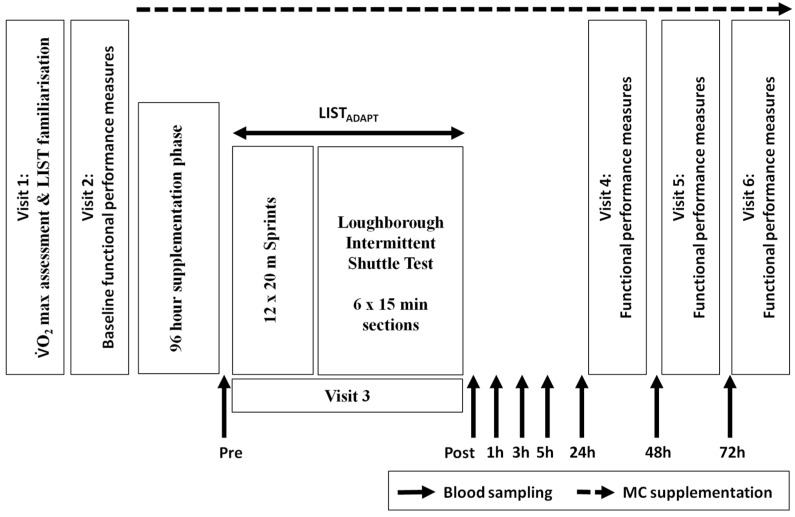
Timeline of study protocol including visit requirements, sampling point and supplementation period.

A double blind, placebo controlled design with independent groups design was employed. Participants attended the laboratory on six separate occasions across a period of no longer than 15 days (Figure 1). On visit 1, participants completed a multi-stage shuttle test [23] in order to predict VO_2max_, which was followed by familiarisation with a battery of functional performance tests and one 15 min section of the Loughborough Intermittent Shuttle Test Part A (LIST) [6]. Participants were then randomly but equally assigned to either a Montmorency cherry (MC) or placebo (PLA) group, matched by predicted VO_2max_ score (54.3 vs. 55.4 mL·kg^−1^·min^−1^).

Participants returned to the laboratory for visit 2 within 5 days to complete the battery of baseline functional measures that followed a standardised warm up; these were countermovement jump height (CMJ), 20 m sprint time (20 m), MVIC of the knee extensors, agility (5-0-5), which were preceded by assessment of active muscle soreness (DOMS). Participants were then provided with 24 MC or PLA beverages in a double blind manner along with verbal and written instructions on how to consume the beverages. They were also provided with a diet record diary and a list of foods to avoid throughout the 4 days prior to and during the trial period. During the 4 day period leading up to the trial day, participants were contacted and instructed to begin supplementation and dietary restrictions.

Visits 3–6 commenced at 8:00 a.m. in order to account for diurnal variation. On visit 3, participants were required to complete an adapted version of the LIST (LIST_ADAPT_). Following the standardised warm-up, participants completed a series of 12 × 20 m sprints with a 10 m ‘stopping zone’, departing every 60 s. This addition was included because (1) the LIST protocol does not account for the many bounding, leaping and directional changes that are associated with team sports play; and (2) previous work from our laboratory has shown only moderate responses with regards to the magnitude of stress response following the LIST [9]. A secondary adaptation to the LIST protocol was the completion of 6 × 15 min sections from the LIST Part A, as opposed to 5 × 15 min sections detailed in the original protocol [6]. This section was included to standardise the distance covered by the two groups (in the original protocol, LIST Part B required a run to exhaustion, potentially resulting in group differences). During the LIST_ADAPT_ participants were provided with water ad libitum.

Visits 4–6 took place at 24, 48 and 72 h following the start of visit 3 and required participants to complete the functional performance test battery outlined in visit 2. Venous blood samples were collected at baseline (prior to muscle soreness and performance test), immediately pre-trial, immediately post-trial and 1, 3, 5, 24, 48 and 72 h post-trial for markers of inflammation, oxidative stress and muscle damage.

### 2.3. Supplementation 

The MC or PLA supplementation was provided to participants after the initial visit along with instructions detailing the dosing schedule (30 mL twice per day, (8:00 a.m., 6:00 p.m.), 7 consecutive days (4 days pre- and on each trial day [13])). Supplements were prepared by mixing each dose with 100 mL of water prior to consumption. The MC was a commercially available Montmorency cherry concentrate (CherryActive, Sunbury, UK); previous work from our laboratory has shown that the MC used in this study contains a total anthocyanin content of 73.5 mg·L^−1^ of cyanidin-3-glucoside, a total phenolic content of 178.8 gallic acid equivalent·L^−1^ and an antioxidant capacity (TEAC) of 0.58 trolox equivalents·L^−1^ [24]. A commercially available, less than 5% fruit, cordial, mixed with water and maltodextrin (MyProtein Ltd, Northwich, UK) until matched for energy content of the MC (102 kcal) was used for the PLA supplement. All supplements were prepared by an independent member of the department prepared in opaque bottles in order to maintain the double blind design.

### 2.4. Dietary and Exercise Restrictions

Participants were instructed to follow a low polyphenolic diet in the 48 h prior to the beginning of each MC or PLA supplementation and throughout the experimental phase of each study. A list of foods to avoid was provided and compliance was assessed through the completion of daily food diaries which has been successfully implemented in previous research [25]. This control measure was used to provide a washout period of polyphenols to enable the efficacy of the phenolic-rich cherry concentrate intervention. In addition, participants were instructed to abstain from any exercise that was not a part of the protocol, throughout the same time periods. Lastly, participants attended all exercise trials following an overnight fast. These measures ensured that dependant variable changes from baseline were likely to be in response to the supplementation and the exercise trials implemented within each study.

### 2.5. Functional Performance Tests

The functional performance test battery was performed in the following order on each occasion: Active muscle soreness assessment (DOMS), 20 m sprint, 5-0-5 agility (CV 2.8% [26]), countermovement jump (CV 1.9%), knee extensors (repeatability in Chapter 3.4). Timings were kept consistent throughout all functional performance tests, each test was performed 3 times (excluding DOMS), with a 1 min rest between repetitions and 3 min rest between tests.

Delayed onset of muscle soreness (DOMS) in the lower limbs was assessed using a 200 mm visual analogue scale (VAS) with ‘no soreness’ at one end and ‘unbearably painful’ at the other. On each occasion the VAS was used, participants were instructed to place their hands on hips, squat down to ~90°, before standing up and immediately making a mark on the scale consistent with their perceived soreness. 

Sprint performance (20 m; coefficient of variation (CV) 0.9%) and the 5-0-5 agility test [27,28] (CV 2.8%) were assessed using wireless telemetry and infra-red timing gates (Brower Timing Systems, Draper, UT, USA) on an indoor athletics track. Countermovement jump height (CV 1.9%) was assessed using a jump mat (Just Jump, Probotics Inc., Huntsville, AL, USA); participants were instructed to stand on the jump mat with their feet parallel and approximately shoulder width apart. Following this, participants completed a maximal vertical jump whilst maintaining hands on hips through flight and landing. MVIC of the dominant knee extensors was determined using a strain gauge (MIE Medical Research Ltd., Leeds, UK) using the methods described previously [13]. The peak performance from each trial was used for data analysis.

### 2.6. Blood Sampling

Blood samples (35 mL) were collected from a forearm vein located in the antecubital fossa region in order to assess for markers of muscle damage (creatine kinase [CK]), inflammation (interleukin-1-beta (IL-1-β), interleukin-6 (IL-6), tumour necrosis factor-alpha (TNF-α), high-sensitivity C-reactive protein (hsCRP)) and oxidative stress (lipid hydroperoxides (LOOH)) using previously described methods [12,13]; intra-sample CVs ranged from 0.7% to 6.8%. Samples were collected into serum gel, ethylenediaminetetraacetic acid (EDTA) or sodium heparin treated tubes (Vacutainer^®^BD UK Ltd., Oxford, UK). Samples were then immediately centrifuged (Allegra X-22 Centrifuge, Beckman Coulter, Bucks, UK) at 2400× *g* at 4 °C for 15 min before having the supernatant removed and stored in aliquots. Aliquots were then immediately stored at −80 °C and subsequently analysed for the respective indices in each study. 

### 2.7. Statistical Analysis

All data analyses were conducted using IBM SPSS Statistics 20 for Windows (Surrey, UK) and are reported as mean ± standard deviation. All data was confirmed as parametric via a Shapiro-Wilk test for normality. Differences between blood marker variables were analysed by using a group (MC vs. PLA) by time-point (Pre-supplement, Post-supplement, 1, 3, 5, 24, 48 and 72 h) mixed model ANOVA. Functional performance measures were analysed using the same model, however with four fewer levels (Pre-supplement, Post-supplement, 24, 48 and 72 h). Where significant group baseline differences were apparent (MVIC, CMJ, 5-0-5 agility, 20 m sprint, DOMS) results were normalised to baseline values prior to subsequent statistical analysis. Mauchley’s Test of Sphericity was used to assess homogeneity of data and where violations were present, Greenhouse-Geiser adjustments were made. Where necessary, interaction effects were assessed using LSD post hoc analysis. Prior to all analyses, a significance level of *p* < 0.05 was set.

## 3. Results

**Figure 2:**
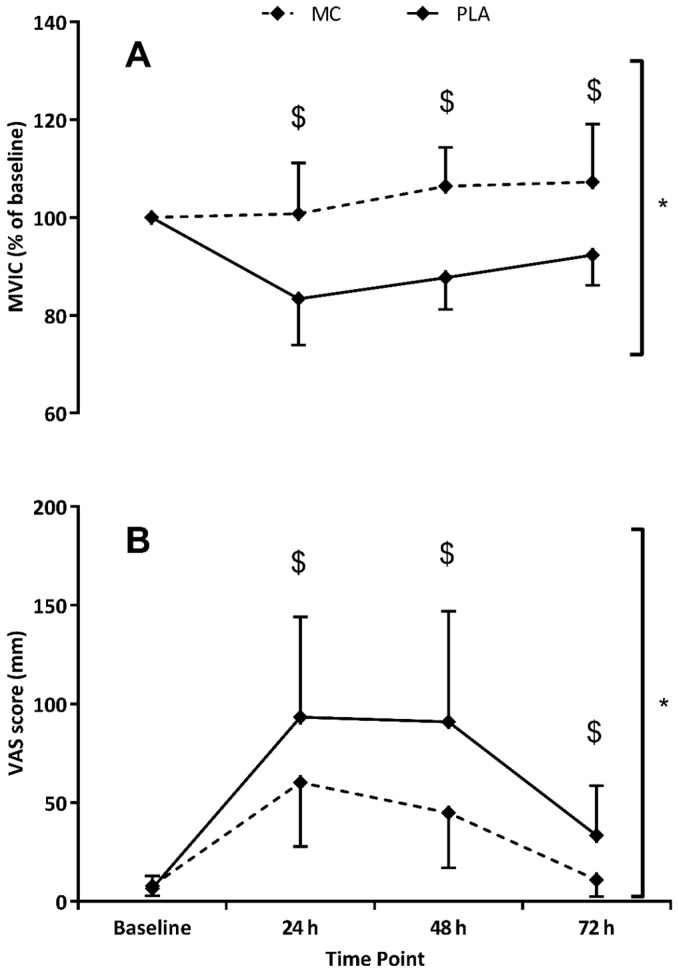
Changes in maximum voluntary isometric contraction (MVIC; Panel (**A**)) and delayed onset muscle soreness (DOMS; Panel (**B**)) in response to Montmorency cherry (MC) or placebo (PLA) supplementation. * Group effect; ^$^ Interaction effect (*p* < 0.05).

MVIC (Figure 2) showed significant time (*F*_(1,4)_ = 6.586, *p* = 0.001, $\eta^{2}$ = 0.320), group (*F*_(1,2)_ = 19.445, *p* = 0.001, $\eta^{2}$ = 0.582) and interaction (*F*_(1,4)_ = 8.970, *p* < 0.001, $\eta^{2}$ = 0.391) effects when data was normalised to baseline values. The decline in MIVC performance was not evident in the MC group whereas as function had not returned to basal levels at 72 h in the PLA. The peak difference occurred at 48 h where MVIC scores in the MC group were found to be 19% higher.

**Figure 3:**
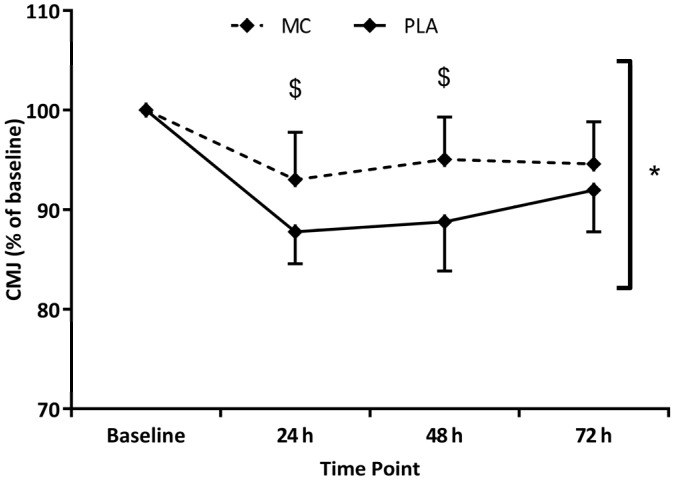
Comparison of countermovement jump (CMJ) height with Montmorency cherries (MC) or placebo (PLA) supplementation. * Group effect; ^$^ Interaction effect (*p* < 0.05).

When data was normalised to baseline values, CMJ also showed significant time (*F*_(1,4)_ = 30.320, *p* < 0.001, $\eta^{2}$ = 0.684), group (*F*_(1,2)_ = 7.336, *p* = 0.017, $\eta^{2}$ = 0.345) and interaction (*F*_(1,4)_ = 3.334, *p* = 0.028, $\eta^{2}$ = 0.193) effects (Figure 3). Both MC and PLA groups demonstrated reduced CMJ (vs. baseline) in the 72 h post-trial period, although the CMJ decrease in the MC group was significantly attenuated at 24 h (5%, *p* = 0.022) and 48 h (6%, *p* = 0.017) versus placebo. Significant time (*F*_(1,4)_ = 12.988, *p* < 0.001, $\eta^{2}$ = 0.481) and group (*F*_(1,2)_ = 7.963, *p* = 0.015, $\eta^{2}$ = 0.355) effects were found for the 5-0-5 agility test. MC times for the 5-0-5 agility were on average 3% faster (vs. PLA) across the 72 h post-trial testing period. For the last of the performance measures, 20 m sprint time, significant time (*F*_(1,4)_ = 9.681, *p* = 0.001, $\eta^{2}$ = 0.409) and interaction (*F*_(1,4)_ = 3.145, *p* = 0.035, $\eta^{2}$ = 0.183) effects were apparent, with both MC and PLA groups demonstrating slower times in all three post-trial tests. At 48 h in the MC group, 20 m sprint times were significantly (*p* = 0.043) faster (4%) than PLA. DOMS was significantly increased in both groups across the 72 h post-trial period (*F*_(1,4)_ = 37.206, *p* < 0.001). A significant group effect (*F*_(1,2)_ = 8.486, *p* = 0.011, $\eta^{2}$ = 0.377) showed that DOMS ratings were lower in the MC group (vs. PLA), which were mirrored by interaction effects (*F*_(1,4)_ = 4.069, *p* = 0.013, $\eta^{2}$ = 0.225) at 24 (*p* = 0.044), 48 (*p* = 0.018) and 72 h (*p* = 0.007), which showed almost complete recovery of at 72 h.

**Figure 4:**
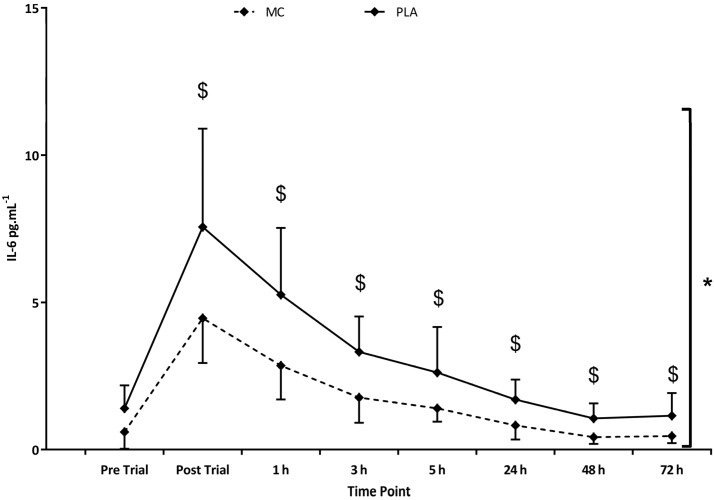
Interleukin-6 (IL-6) responses in the Montmorency cherry (MC) and placebo (PLA) groups to the adapted Loughborough Intermittent Shuttle Test (LIST_ADAPT_) exercise. * Group effect; ^$^ Interaction effect (*p* < 0.05).

With regards to inflammatory markers, IL-6 (Figure 4) was found to be elevated in both groups following the trial (*F*_(1,8)_ = 52.180, *p* < 0.001, $\eta^{2}$ = 0.788). Group comparisons (*F*_(1,2)_ = 10.223, *p* = 0.006, $\eta^{2}$ = 0.422) demonstrated an overall significantly attenuated IL-6 response to the trial in MC (vs. PLA), with significant interaction effects (*F*_(1,8)_ = 3.313, *p* = 0.003, $\eta^{2}$ = 0.191) showing peak differences of 3.10 pg·mL^−1^ occurring immediately post-exercise (*p* = 0.03). Further inflammatory marker data for plasma IL-8 (*F*_(1,8)_ = 4.905, *p* = 0.010, $\eta^{2}$ = 0.259), TNF-α (*F*_(1,8)_ = 6.343, *p* < 0.001, $\eta^{2}$ = 0.312) and hsCRP (*F*_(1,8)_ = 20.298, *p* < 0.001, $\eta^{2}$ = 0.592) revealed significant increases in each variable in the 72 h following the trial, however, group and interaction comparisons failed to identify differences. IL-1-β was not found to be increased at any measurement point across the trial period. The trial significantly increased CK in both groups (*F*_(1,4)_ = 10.243, *p* = 0.004, $\eta^{2}$ = 0.423), although no group or interaction effects were found. Peaks values of 1200 IU/L were attained at 24 h (Table 1).

Lipid hydroperoxides were increased in the 72 h post-exercise period as indicated by a significant time effect (*F*_(1,8)_ = 5.973, *p* < 0.001, $\eta^{2}$ = 0.289). Peak increases of 35% above baseline occurred at 5 h. Although there was a tendency for higher PLA group values, no significant group or interaction effects were found. A summary of variables is reported in Table 1.

In order to identify any group differences in LIST_ADAPT_ performance, a comparison of sprint times during the LIST_ADAPT_ protocol was performed using Student’s *T*-test. No group differences in LIST_ADAPT_ sprint performance were found (*t* = 1.511, *p* = 0.153).

## 4. Discussion

The main finding of this study was that participants supplemented with MC were able to maintain greater functional performance than PLA counterparts following prolonged intermittent sprint activity. More specifically, MVIC, CMJ, 20 m sprint and 5-0-5 agility performances were superior in the 72 h post-exercise with MC (vs. PLA). In addition, DOMS and IL-6 were lower in the MC group throughout the post-trial period. 

The attenuated declines in muscle performance are consistent with the findings of previous studies investigating MC as a recovery aid [12,13,14,15,16] and additionally the magnitude of MVIC decline following the LIST_ADAPT_ was comparative to previous work utilising a similar protocol [29]. The MVIC performance was on average 17%, superior in the MC group (vs. PLA). MC supplementation also resulted in better (vs. PLA) CMJ performance at 24 and 48 h. Sprint times in both groups were slower in the 72 h post-exercise period, however at 48 h the MC group was significantly faster. Agility (5-0-5) times were also faster in the MC group by an average of 3% across the 72 h post-trial period. Interestingly, the MVIC results in the 72 h following exercise suggests MC supplementation abolishes declines in this performance measure—a result that has been previously reported [13].

These data support the idea that supplementation with MC protect declines in muscle function following strenuous exercise, specifically in activity akin to repeated sprint sports and games play such as rugby, soccer and field hockey. A reduction in post-exercise IL-6 suggests a lower acute inflammatory response to the exercise bout that might contribute to the performance differences between groups. The COX, prostaglandin, IL-6 pathway, which are activated during the secondary inflammatory response to cellular disruption, has been associated with proteolytic and lipolytic processes [30] and subsequently muscular performance could be inhibited. Seemingly, MC supplementation reduced (but did not abolish) this process and allowed for greater maintenance of muscular performance in the recovery period. Conversely, there were no group differences in hsCRP. This is unexpected given that IL-6 is implicated as a signalling molecule for the expression of hsCRP [31,32]. We are unable to resolve the discrepancy between IL-6 and hsCRP and therefore suggest further work is needed to identify the mechanism by which MC might exert its anti-inflammatory responses in response to strenuous exercise.

In contrast to previous work [12] that examined repeated days cycling exercise, there were no differences in LOOH between groups. The obvious discrepancy between study findings may be attributed to single versus repeated days exercise. Unlike Bell et al. [12], where repeated days cycling exercise (metabolic challenge) were used, the current study investigated a single bout of strenuous exercise that incorporated both a metabolic and mechanical exercise stress. Conceptually, the accumulated stress response from repeated day’s exercise would be greater than a single bout, but of course the modalities, cycling versus simulated concurrent exercise, pose very different exercise challenges and hence the redox response may also differ considerably between exercise stimuli. 

In agreement with previous work [16,22], the lower post-exercise DOMS in the MC group provide further evidence for the protective effect of cherries. Despite this, previous work from our lab [13] using MC has not shown this positive effect. This discrepancy may be attributed to the different modes of exercise employed to induce stress. Whilst muscle actions during cycling are almost exclusively concentric [33], the repeated sprints and decelerations during the LIST_ADAPT_ protocol in the present study, place a heavy eccentric load on the same muscle groups and as a result are likely to incur greater mechanical stress. Indeed, DOMS ratings from the present study were consistently higher than those in the cycling studies [12,13]. In further support of this supposition, CK (an index of cellular disruption following damaging exercise) was considerably higher than the aforementioned cycling studies. However, the CK response reported in previous work [14,15] using protocols that also incorporate a heavy eccentric component, showed no evidence for a protective effect of MC.

The high-intensity, prolonged, intermittent nature of soccer and other repeated sprint sports places a high degree of both mechanical and metabolic stress [2], which is reflected by the increase in the appearance of physiological stress responses in the present study. Whilst this is not the first study to demonstrate accelerated recovery of functional performance with MC supplementation, it is the first to do so following simulated games play and therefore represents an important wider application of this intervention to aid exercise recovery in sports of an intermittent sprint nature, such as soccer, rugby, field hockey and basketball. Collectively, there is a growing body of evidence suggesting that MC has the ability to facilitate exercise recovery—perhaps by modulating inflammation and/or oxidative stress. The exact mechanisms behind these promising data are not clear, so it seems prudent to explore further; perhaps by using animal, cellular and molecular techniques to provide a greater understanding of the application of MC and other phenolic-rich foods.

In summary, this study provides further evidence for the use of MC as a recovery aid. For the first time, MC supplementation has been shown to accelerate the recovery of a number of functional performance measures following prolonged intermittent sprint activity and suggest that some dampening of the post-exercise inflammatory processes might be responsible. With regards to application, the dampening of such responses could be highly advantageous in sports requiring athletes to complete high volumes of training whilst ‘in-season’, or athletes competing in tournament scenario’s that require multiple performances within a short time period and the ability to recover in sufficient time is a challenge. Additionally, although dietary restrictions were imposed throughout the study period, the results suggest that sports requiring sprinting or high intensity directional changes might benefit from MC supplementation when playing schedules are congested and recovery time is limited between games. Finally, the issue of modulating the post-exercise oxidative stress and inflammatory response has raised concerns; insofar as these stressors are implicated in the adaptive response. Although there is no evidence that the adaptive response is affected by functional foods, this question should be addressed in order to determine if periodiation of these sorts of supplements is warranted.

## Figures and Tables

**Table 1:** Summary of other performance, inflammatory and oxidative stress data.

	Pre-Trial		Post-Trial		1 h		3 h		5 h		24 h		48 h		72 h	
	Mean	SD	Mean	SD	Mean	SD	Mean	SD	Mean	SD	Mean	SD	Mean	SD	Mean	SD
5-0-5 Agility (s) *^,$^																
MC	2.34	0.11									2.42	0.17	2.38	0.10	2.35	0.12
PLA	2.30	0.13									2.41	0.17	2.43	0.17	2.37	0.18
20 m Sprint (s) *																
MC	3.11	0.14									3.17	0.15	3.14	0.12	3.12	0.15
PLA	3.05	0.12									3.18	0.18	3.23	0.24	3.17	0.19
DOMS (mm) *^,$^																
MC	8	5									60 ^+^	32	44 ^+^	28	10 ^+^	8
PLA	7	6									93 ^+^	51	90 ^+^	56	33 ^+^	25
IL-1-β (pg·mL^−1^)																
MC	0.03	0.09	0.04	0.08	0.03	0.06	0.02	0.04	0.11	0.13	0.03	0.05	0.06	0.14	0.01	0.01
PLA	0.02	0.04	0.02	0.05	0.36	1.00	0.02	0.05	0.02	0.02	0.01	0.02	0.01	0.04	0.03	0.03
IL-8 (pg·mL^−1^) *																
MC	2.06	0.52	4.37	0.93	3.77	0.87	3.04	1.13	3.29	0.79	2.23	0.53	1.90	0.64	2.18	0.70
PLA	2.28	0.54	4.69	1.78	3.67	0.99	3.03	0.74	3.25	0.86	2.50	0.76	2.25	0.63	2.49	0.92
TNF-α (pg·mL^−1^) *																
MC	1.16	0.34	1.23	0.42	1.18	0.29	1.16	0.32	1.11	0.35	1.36	0.39	1.22	0.38	1.36	0.52
PLA	1.77	0.88	1.96	0.84	1.76	0.69	1.73	0.77	1.60	0.77	1.83	0.90	1.78	0.80	1.88	0.97
CK (IU·L^−1^) *																
MC	293	228	691	365	885	504	1243	1004	1538	1317	1551	1473	959	822	560	386
PLA	197	143	439	310	535	401	744	625	989	946	1034	1096	652	603	403	319
hsCRP (pg·mL^−1^) *																
MC	0.70	0.86	0.70	0.82	0.69	0.83	0.69	0.75	0.90	0.69	1.94	0.88	1.39	0.63	1.00	0.54
PLA	1.24	1.38	1.36	1.36	1.40	1.48	1.40	1.47	1.55	1.60	2.93	2.73	2.29	1.63	1.73	1.22
LOOH (mmol·mL^−1^) *																
MC	1.37	0.10	1.59	0.29	1.37	0.25	1.62	0.34	1.60	0.28	1.25	0.14	1.26	0.16	1.25	0.17
PLA	1.37	0.21	1.61	0.33	1.41	0.27	1.96	1.34	2.15	1.44	1.24	0.15	1.29	0.24	1.23	0.16

* Significant main effect for time (*p* < 0.001); ^$^ group; ^+^ group × time interaction. DOMS, Delayed Onset Muscle Soreness; IL-1-β, Interleukin-1-beta; IL-8, Interleukin-8; TNF-α, Tumour Necrosis Factor-Alpha; CK, Creatine Kinase; hsCRP, high-sensitivity C-Reactive Protein; LOOH, Lipid Hydroperoxides.

## References

[1] Spencer M., Bishop D., Dawson B., Goodman C. (2005). Physiological and metabolic responses of repeated-sprint activities: Specific to field-based team sports. Sports Med..

[2] Ispirlidis I., Fatouros I.G., Jamurtas A.Z., Nikolaidis M.G., Michailidis I., Douroudos I., Margonis K., Chatzinikolaou A., Kalistratos E., Katrabasas I. (2008). Time-course of changes in inflammatory and performance responses following a soccer game. Clin. J. Sport Med..

[3] Andersson H., Bøhn S.K., Raastad T., Paulsen G., Blomhoff R., Kadi F. (2010). Differences in the inflammatory plasma cytokine response following two elite female soccer games separated by a 72-h recovery. Scand. J. Med. Sci. Sports.

[4] Ascensão A., Rebelo A., Oliveira E., Marques F., Pereira L., Magalhães J. (2008). Biochemical impact of a soccer match—Analysis of oxidative stress and muscle damage markers throughout recovery. Clin. Biochem..

[5] Fatouros I.G., Chatzinikolaou A., Douroudos I.I., Nikolaidis M.G., Kyparos A., Margonis K., Michailidis Y., Vantarakis A., Taxildaris K., Katrabasas I. (2010). Time-course of changes in oxidative stress and antioxidant status responses following a soccer game. J. Strength Cond. Res..

[6] Nicholas C.W., Nuttall F.E., Williams C. (2000). The loughborough intermittent shuttle test: A field test that simulates the activity pattern of soccer. J. Sports Sci..

[7] Cockburn E., Bell P.G., Stevenson E. (2013). Effect of milk on team sport performance following exercise-induced muscle damage. Med. Sci. Sports Exerc..

[8] Magalhaes J., Rebelo A., Oliveira E., Silva J.R., Marques F., Ascensao A. (2010). Impact of loughborough intermittent shuttle test versus soccer match on physiological, biochemical and neuromuscular parameters. Eur. J. Appl. Physiol..

[9] Leeder J., van Someren K.A., Gaze D., Jewell A., Deshmukh N., Shah I., Howatson G. (2014). Recovery and adaptation from repeated intermittent-sprint exercise. Int. J. Sports Physiol. Perform..

[10] Reilly T., Ekblom B. (2005). The use of recovery methods post-exercise. J. Sports Sci..

[11] Russell M., Northeast J., Atkinson G., Shearer D.A., Sparkes W., Cook C.J., Kilduff L. (2015). The between-match variability of peak power output and creatine kinase responses to soccer match-play. J. Strength Cond. Res..

[12] Bell P.G., Walshe I.H., Davison G.W., Stevenson E., Howatson G. (2014). Montmorency cherries reduce the oxidative stress and inflammatory responses to repeated days high-intensity stochastic cycling. Nutrients.

[13] Bell P.G., Walshe I.W., Davison G.W., Stevenson E.J., Howatson G. (2014). Recovery facilitation with montmorency cherries following high-intensity, metabolically challenging exercise. Appl. Physiol. Nutr. Metab..

[14] Howatson G., McHugh M.P., Hill J.A., Brouner J., Jewell A.P., Van Someren K.A., Shave R.E., Howatson S.A. (2010). Influence of tart cherry juice on indices of recovery following marathon running. Scand. J. Med. Sci. Sports.

[15] Bowtell J.L., Sumners D.P., Dyer A., Fox P., Mileva K. (2011). Montmorency cherry juice reduces muscle damage caused by intensive strength exercise. Med. Sci. Sports Exerc..

[16] Connolly D.A.J., McHugh M.P., Padilla-Zakour O.I. (2006). Efficacy of a tart cherry juice blend in preventing the symptoms of muscle damage. Br. J. Sports Med..

[17] McCune L.M., Kubota C., Stendell-Hollis N.R., Thomson C.A. (2011). Cherries and health: A review. Crit. Rev. Food Sci. Nutr..

[18] Bell P.G., Gaze D.C., Davison G.W., George T.W., Scotter M.J., Howatson G. (2014). Montmorency tart cherry (prunus cerasus l.) concentrate lowers uric acid, independent of plasma cyanidin-3-*O*-glucosiderutinoside. J. Funct. Foods.

[19] Keane K.M., Bell P.G., Lodge J.K., Constantinou C.L., Jenkinson S.E., Bass R., Howatson G. (2016). Phytochemical uptake following human consumption of montmorency tart cherry (L. *Prunus cerasus*) and influence of phenolic acids on vascular smooth muscle cells in vitro. Eur. J. Nutr..

[20] Wang H., Nair M.G., Strasburg G.M., Chang Y.-C., Booren A.M., Gray J.I., DeWitt D.L. (1999). Antioxidant and antiinflammatory activities of anthocyanins and their aglycon, cyanidin, from tart cherries. J. Nat. Prod..

[21] Seeram N.P., Momin R.A., Nair M.G., Bourquin L.D. (2001). Cyclooxygenase inhibitory and antioxidant cyanidin glycosides in cherries and berries. Phytomedicine.

[22] Kuehl K., Perrier E., Elliot D., Chesnutt J. (2010). Efficacy of tart cherry juice in reducing muscle pain during running: A randomized controlled trial. J. Int. Soc. Sports Nutr..

[23] Léger L.A., Lambert J. (1982). A maximal multistage 20-m shuttle run test to predict VO2 max. Eur. J. Appl. Physiol. Occup. Physiol..

[24] Keane K.M., George T.W., Constantinou C.L., Brown M.A., Clifford T., Howatson G. (2016). Effects of montmorency tart cherry (*Prunus cerasus* L.) consumption on vascular function in men with early hypertension. Am. J. Clin. Nutr..

[25] Howatson G., Bell P.G., Tallent J., Middleton B., McHugh M.P., Ellis J. (2012). Effect of tart cherry juice (*Prunus cerasus*) on melatonin levels and enhanced sleep quality. Eur. J. Nutr..

[26] Sayers M., Kilip J.V. Reliability and validity of the 5-0-5 agility test. Proceedings of the 4th Evolution of the Athlete Coach Education Conference.

[27] Davies V., Thompson K.G., Cooper S.M. (2009). The effects of compression garments on recovery. J. Strength Cond. Res..

[28] Houghton L.A., Dawson B.T., Rubenson J. (2013). Effects of plyometric training on achilles tendon properties and shuttle running during a simulated cricket batting innings. J. Strength Cond. Res..

[29] Leeder J.D., Van Someren K.A., Bell P.G., Spence J.R., Jewell A.P., Gaze D., Howatson G. (2015). Effects of seated and standing cold water immersion on recovery from repeated sprinting. J. Sports Sci..

[30] Trappe T.A., Standley R.A., Jemiolo B., Carroll C.C., Trappe S.W. (2013). Prostaglandin and myokine involvement in the cyclooxygenase-inhibiting drug enhancement of skeletal muscle adaptations to resistance exercise in older adults. Am. J. Physiol. Regul. Integr. Comp. Physiol..

[31] Petersen A.M., Pedersen B.K. (2005). The anti-inflammatory effect of exercise. J. Appl. Physiol..

[32] Pepys M.B., Hirschfield G.M. (2003). C-reactive protein: A critical update. J. Clin. Invest..

[33] Bijker K., de Groot G., Hollander A. (2002). Differences in leg muscle activity during running and cycling in humans. Eur. J. Appl. Physiol..

